# Diet Education as a Success Factor of Glycemia Regulation in Diabetes Patients: A Prospective Study

**DOI:** 10.3390/ijerph16204003

**Published:** 2019-10-19

**Authors:** Zvjezdana Gvozdanović, Nikolina Farčić, Harolt Placento, Robert Lovrić, Željka Dujmić, Ana Jurić, Blaženka Miškić, Nada Prlić

**Affiliations:** 1 General Hospital Našice, Našice 31 500, Croatia; zvjezdana.gvozdanovic@obnasice.hr (Z.G.); harolt.placento@obnasice.hr (H.P.); ana.juric.liovic@gmail.com (A.J.); 2 Faculty of Medicine, Josip Juraj Strossmayer University of Osijek, Osijek 31 000, Croatia; 3 Nursing Institute “Professor Radivoje Radić”, Faculty of Dental Medicine and Health Osijek Josip Juraj Strossmayer University of Osijek, Osijek 31 000, Croatia; rlovric@mefos.hr (R.L.); dujmic.zeljka@gmail.com (Ž.D.); nadaprlic26@gmail.com (N.P.); 4 Department of Surgery, University Hospital Centre Osijek, Osijek 31 000, Croatia; 5 General Hospital “Dr. Josip Benčević” Slavonski Brod, Slavonski Brod 35 000, Croatia; blazenka.miskic@bolnicasb.hr

**Keywords:** diabetes mellitus, diet, diabetes education, glycemia regulation

## Abstract

Background: The aim of this study was to examine the effect of dietary education on glycemic control in patients with any type of diabetes at four-week and two-year follow-ups. Methods: A two-year prospective study was conducted in three phases: before, four weeks after, and two years after an educational program. The participants were patients diagnosed with diabetes who were receiving insulin or oral hypoglycemics and who attended the Diabetes Clinic of the General County Hospital Našice, Croatia to receive their treatment. The questionnaire and educational program were created for the purpose of this study. Measurements were made (fasting blood glucose, glycated hemoglobin, body mass index, blood pressure, knowledge test) at baseline, four weeks and two years after education. Results: A total of 109 participants took part in the study, of which 56 (51.4%) were on insulin therapy and 53 (48.6%) were on oral therapy, after two years, 78 (72%) subjects were followed up with. There was no control group. At the two-year follow-up, participants with insulin therapy had significantly higher glycated hemoglobin (Mann–Whitney U test, *p* = 0.035). Significant differences were observed in fasted blood glucose (Friedman’s test, *p* = 0.001), diastolic pressure (Friedman’s test, P = 0.018), and glycated hemoglobin (Wilcoxon test, *p* < 0.001) between Phase 1 and Phase 3. Conclusions: Education has a positive effect on adherence to recommended diet and glycemia regulation in diabetes patients after four-week follow-ups. However, after two years, participants showed a decrease in adherence to recommended diet and increased glycemia.

## 1. Introduction

Today, chronic non-communicable diseases are the leading cause of mortality and hospitalization and represent a major public health problem [1]. The number of people diagnosed with diabetes continues to grow annually [2]. Type 2 diabetes is caused by the lifestyle change of habits of most of the population such as poor diet and nutrition, obesity, physical inactivity, and stress [2]. According to estimates by the International Diabetes Federation, the prevalence of diabetes in 2017 was 8.8% worldwide and 6.8% in Europe among people aged 20–79 years [2]. In addition, this incidence is projected to steadily increase [2]. Croatia fits with the global incidence, according to the data of the Croatian Institute for Public Health, the prevalence of diabetes in 2015 was 8.34% in Croatia among people aged 20–79 years, and the total number of registered patients with diabetes was 268,565 [3]. About 1 in 11 adults worldwide now suffer from diabetes mellitus, 90% of whom suffer from type 2 diabetes mellitus [1]. Data analysis of CroDiab register generally does not point to significant deviations in certain instances share of type I, type II, and other types of diabetes [4]. In Croatia, the share of type I diabetes ranges from 6.5% and 7.5%, type II ranges between 90% and 93%, and other types of diabetes vary from 0.5% to 1.5% [4]. 

Best management practices encourage patients to measure their blood glucose, take medications, and adjust their dietary habits to maintain health with the disease [5]. Because outcomes are largely dependent on decisions made by the patients, patients with diabetes should receive on-going and high-quality education adapted to their needs by qualified health care professionals [5]. Diabetes educators are one of the contributors in providing individualized education and promoting behavior change, they are health care professionals who apply in-depth knowledge and skills in the medical and social sciences, pedagogy, and communication to enable patients to manage daily and future challenges [6]. 

The overall goal of diabetes education is to help individuals and their families gain the necessary knowledge, life skills, resources, and support needed to achieve optimal health [7]. When planning health educational programs, it is necessary to evaluate what content should be included by considering the needs of the people who will be educated [8]. Investments in diabetes education and prevention are expected to save money long-term and improve the quality of life of patients with diabetes [8]. The role of a diabetes nurse specialist is an integral part of education which enables improving diabetes care, avoiding hospital admissions [9], and reducing the length of a hospital stay [10]. The most common nursing interventions to patients with diabetes are nutritional counseling, exercise promotion, education about feet care, control of hypoglycemia and hyperglycemia, and prescription medicines [11]. Patients who are educated about the disease and remain motivated require fewer visits to a doctor, miss less time from work or school, and better recognize acute situations associated with this chronic illness [12]. The aim of this study was to examine the effect of dietary education on glycemic control and adherence to diabetic diet in patients suffer from diabetes at four-week and two-year follow-ups and to compare the effect of dietary education four weeks and two years after the education. 

## 2. Materials and Methods 

Prospective research regarding “knowledge questionnaire” was conducted in order to validate it. Research was conducted in three phases: Phase 1 from 10 June 2014 to 14 August 2014, Phase 2 which was conducted four weeks after the phase 1 depending on the beginning of the Phase 1, and Phase 3 which was conducted two years after the Phase 2, from 5 August 2016 to 13 October 2016. 

The educational program was administered by a team of diabetes specialist nurses, internal specialists, and diabetes experts. The education was conducted individually and in small groups. The group received the required education within five two-hour sessions in groups of 3–4 individuals in the period of one week and one session individually. Before the education program, participants completed a questionnaire; and fasting blood glucose, glycated hemoglobin, body mass index, blood pressure measurements were performed in a fasted state. The results obtained from these measurements were the basis for the development of the educational program. The education was individualized based on the questionnaire results (small groups), but it was similar to all of the participants. Four weeks after the education (Phase 2), subjects completed the knowledge test again using the same questionnaire, and measurements were repeated. Two years after the education, subjects were contacted by telephone to attend a follow-up meeting where they completed the questionnaire a third time, and the measurements were repeated (Phase 3).

The study was conducted at the Diabetes Clinic of the General County Hospital Našice, which is a general hospital in Osijek-Baranja County, Croatia. The population gravitating to our hospital is 16,000, primarily rural.

The inclusion criteria were male or female patients with any type of diabetes who were receiving insulin, oral hypoglycemics, or combination therapy of insulin and oral hypoglycemics, having at least one treatment prior to the study, with an active record in the diabetes clinic and informed consent to participate in the research. The exclusion criteria were patients under 18 and/or over 75 years of age, cognitive and/or mental diseases, illiteracy, patients with life expectancy of less than 2 years, and the patients who did not participate in educational sessions (less than 60% of sessions).

### 2.1. Study Tools

#### 2.1.1. Knowledge Questionnaire

As a study tool, a questionnaire was created for the purpose of this study according to the literature on educating and training diabetic patients [13,14]. The first group of questions contained general demographic determinants (3 questions), and the second question group examined the knowledge (15 questions), physical activity (3 questions), self-control (13 questions), and diet (4 questions) of patients suffer from diabetes. Regarding the sample of participants in this study, the reliability of the internal consistency (Cronbach’s alpha) amounts to 0.89 for the whole scale.

#### 2.1.2. Educational Program

Diabetes education was based on the pre-test of diabetic patients and literature on educating and training of diabetic patients and teaching about the illness, possible complications, diabetes diet, taking medications, physical activity, risk factors and their effect on improving treatment outcomes, quality of life and self-management [13,14]. Teaching methods were lecture, discussion and demonstration, on each session. After the education, a diabetes nurse specialist was available by telephone for 1 month to respond to their questions, help, and to fix their problems. In this period, almost 70% of the patients were contacted, once or several times, by a diabetes nurse specialist. Participants were divided into groups as they were included into the research, according to therapy types. The initial session was performed at the point of inclusion and it included receiving information on the research, signing informed consent, filling the questionnaire and motivational conversation on adhering to the recommended diet and therapy. The education lasted one week (five two-hour educational sessions) in the form of daily hospital visits. 

The content and plan used in five two-hour educational sessions included: (1) Patho-physiological view: diabetes definition, range of blood glucose levels, physical activity, and stress. (2) Diet and food: personalized nutritional advice was given based on preferences, but the overall aim was to reduce energy, portions, carbohydrates, fats, salts, and also education aimed at identifying healthy food and amount of energy per gram of carbohydrates, fats, proteins, and quantitative features of recommended diet. (3) Treatment: insulin and oral hypoglycemics administration. (4) Measurements: Level of blood glucose monitoring. (5) Information of acute complications: prevention and treatment of hypoglycemia and hyperglycemia. (6) Information about chronic complications: prevention of late stage chronic complications, prevention of cardiovascular disease and events (myocardial infarction, stroke, and peripheral vascular disease), burden of overweight, the need of smoking cessation, hypertension, and dyslipidemia. (7) Practical tips: source and storage of insulin, monitoring of glucose supplies, diabetes nurse specialist telephone number. Within each session, the participants were offered lunch and participated in practical work by calculating food energy value and identifying recommended food. Recommended diet is suitable for the whole family, the main defining points are low energy, portion, carbohydrate, fat, and salt. Men do not usually buy food or prepare meals, but their wives have not been involved to the education sessions. Participants were provided with written educational content. (Lists of foods that increase blood glucose, recommended foods, food recipes).

In total, 109 patients participated in the first two phases (before and four weeks after the education program), insulin therapy, and combination insulin and oral hypoglycemic (*N* = 56), and oral hypoglycemic (*N* = 53). In the third phase 3/4 patients participated, a total of 78 (72%) (two years after the education program), insulin therapy, and combination insulin and oral hypoglycemic (*N* = 38), and oral hypoglycemic (*N* = 40). Of original subjects, 11 (10%) died, 7 (6.4%) changed their place of residence, 7 (6.4%) declined to participate in the follow-up, and 6 (5.6%) could not be contacted. Some subjects 4 (3.6%) who previously used oral hypoglycemics were transferred to insulin therapy, their medication had changed since their initial involvement in the study. The study was conducted in accordance with the ethical principles and human rights standards of medical study. For the purpose of conducting the investigation, the consent of the Commission for Ethical Affairs of the County General Hospital Našice was obtained (Order No. 01-421/1-2014).

### 2.2. Statistical Analysis 

To make comparison between patients on oral hypoglycemics and those requiring insulin, statistical significance was set at 0.05, a power of 0.8 with an effect of a 0.7, the minimum size needed for the statistical sample was 45 participants per group, i.e., the total sample size was 90 participants (G*Power 3.1.9.2). Considering that the study had a two-year follow-up, the high mortality rate in diabetic patients [1,2], the rural setting, and the advanced age of most patients, our sample intentionally had 20% more participants (109 participants). Categorical and continuous variables were analyzed using absolute and relative frequencies. The numerical data were described by the median and the limits of the interquartile range. The variance of the category variables was tested using hi-quadratic test and the Fisher exact test for small numbers. For continuous variables normal distributions were examined and variables logged if not normally distributed. Normality of the distribution of numeric variables was tested by the Shapiro–Wilk test. Differences in numeric variables between groups were tested by Mann–Whitney U test, Wilcoxon or Friedman’s test. For the study of dependent variables (before and after education), Bowker’s symmetry test was used, which is identical for two categories to the McNemar test and the marginal homogeneity test of the contingency dimension k × k, k > 2 (Bhapkar test). All P values were two-sided. The statistical analysis was performed using the statistical program SPSS Inc. (released 2008, SPSS Statistics for Windows, Version 17.0. Chicago: SPSS Inc).

## 3. Results

### 3.1. Socio-Demographic Characteristics

A total of 109 participants took part in the study, after two years, 78 (72%) subjects were followed up with. The mean age of participants was significantly higher in subjects on oral therapy (Mann–Whitney test, *p* = 0.046). The mean duration of the disease was 8 years (interquartile range, 4–16 years) and was significantly shorter in the oral therapy group (Mann–Whitney test, *p* = 0.006). A majority of participants had completed secondary education qualifications (*n* = 54, 49.5%) (Table 1).

### 3.2. Values of Biometric Indices

Significant differences were observed in fasted blood glucose (Friedman’s test, *p* = 0.001), diastolic pressure (Friedman’s test, *p* = 0.018), and glycated hemoglobin (HbA1c) (Wilcoxon test, *p* < 0.001) between Phase 1 and Phase 3. Regarding systolic pressure and the body mass index, there were no significant differences.

After education, no significant changes in blood glucose, blood pressure, and body weight indexes existed according to therapy, before and after the education. At two-year follow-up, participants with insulin therapy had significantly higher HbA1c (Mann–Whitney U test, *p* = 0.035) (Table 2.)

### 3.3. Adherence to Recommended Diet

There was a difference in the number of patients who maintained a recommended diet before and after the education. Over half of the patients on insulin were able to adhere to the dietary recommendation after education. At two-year follow-up, 9 (24.3%) patients using insulin always followed dietary guidelines and 11 (27.5%) patients taking medications always followed dietary guidelines (McNemar–Bowker test, *p* = 0.048) (Table 3).

### 3.4. Knowledge on Foods that Increase Blood Glucose

There was a significant difference in the knowledge on foods that increase blood glucose before and after education for patients on insulin and patients on oral hypoglycemics therapy. At two-year follow-up, a significant difference was present only in patients using insulin (Table 4).

## 4. Discussion

This study reports on a hospital outpatient-based education program designed to address gaps in knowledge in people with diabetes. Patients were grouped and compared for baseline biographical characteristics depending on whether they were prescribed oral hyperglycemic medications or insulin. Patient completed questionnaires on knowledge and self-reported behaviors before the education sessions. Pre- and post-knowledge, and adherence to a recommended diet comparison were made at 4 weeks and 2 years. Significant improvements were noted for HbA1c, fasting serum glucose, for all patients. However, a significant increase in diastolic blood pressure was found for patients who had prescribed insulin therapy which may be related to their non-significant increase in body mass index (BMI). In addition, significant improvements were found in the proportion of patients who adhered to the recommended diet and for knowledge of food that increases serum glucose levels. However, after two years, participants showed a decrease in adherence to recommended diet and increased glycemia.

Several studies have demonstrated how diet education improved patients’ knowledge and helped them adopt healthier behaviors [15,16,17,18,19,20,21,22,23]. In this study, after the education, participants adhered to a recommended diet and meal planning significantly more and had better knowledge regarding foods that increase blood glucose. These results clearly point to the positive effect of education on the knowledge and behavior of the participants in this study. A similar study carried out in Brazil also showed a significant increase in the level of knowledge of subjects about their disease, particularly regarding knowledge on nutrition [15]. Education contributes to more effective glycemia control and reduces disease complications [16]. Self-care knowledge levels were found to be significantly associated with previous health education about diabetes [17]. Research shows that health education of diabetic patients is crucial for control of diabetes [18]. In contrast, one study found that education had no effect on motivation or knowledge for diabetes patients [24]. Another study carried out in India found that repeated community engagement was effective in diabetes education [19], a group-based peer-support program and group identified peer leaders among themselves, based on their social credibility and willingness to lead the group. After education, the leaders conducted the education of their group with the support from experts. At the end of 12 months, 55% participants found the group sessions ‘very useful’ in making lifestyle changes [19].

To test effectiveness of the systematic health education model for type 2 diabetes mellitus (T2DM), a study was conducted in China. The objectives were accomplished through systematic health education in a group provided by health educators every month for over 2 years. The researchers concluded that the systematic health education model is a useful method in the treatment of DM because it contributes to a decrease in HbA1c (educational group −0,95; control group −0.38), and BMI did not change [20]. In this study after 5 education sessions and a long follow up without education the reduction in HbA1c (insulin group −1.0, oral hypoglycemic group −1.3 = −1.15; no control group) is higher but slight.

The study carried out in Spain showed a reduction in HbA1c of −0.03 after a 2-year follow-up period in interventional group and nonsignificant increase in control group 0.04 [21]. The two groups studied according to the type of health promotion model and they were observed; interventions were 10 visits (both groups) for 2 year (one every 3 months) and individual health education extra 40 minutes per session, while usual proceedings took an extra 20 minutes [21]. These results show the positive effect of education in the overall treatment in patients with diabetes, especially in reduction of HbA1c. The decreased levels of diastolic blood pressure were nonsignificant, and BMI did not change [21]. 

Regarding systolic pressure, there were no significant differences in this study. The study in China showed a decrease of systolic pressure (10.83 mmHg) [20], while this study showed an increase in diastolic pressure (10 mmHg), which may be related to a non-significant increase in BMI. Improvement of living standards, changes in nutrition, and physical inactivity may explain difficulty in reducing BMI. In contrast, in the study conducted in Brazil the BMI decreased significantly when compared with the baseline, up to one year [22]. Participants were randomized to attend an 8-hour structured educational program, delivered in 4 sessions, for 4 weeks, by a trained nurse educator or to usual care. Measurements were made (HbA1c, BMI, fasting blood glucose, blood pressure, knowledge test) at baseline 1, 4, 8 months and 1 year after education. However, the improvement lasted for up to one year, losing momentum after 4 months, and they concluded that education should be part of the routine care of diabetic patients, and it should be repeated periodically, every eight to twelve months [22]. A study in Italy demonstrated that BMI decreased (−1.4) over 5 years among group care after periodically repeated education for 5 years [23]. 

Nurses play an important role in pharmacological and non-pharmacological treatment of diabetic patients [25]. Apart from the individualized education of patients, the review of the diabetic studies recommends the education of health care staff and setting up of a nutritional algorithm that would guide patients through the meal planning process and help them determine the optimal macro-nutritional system [26]. Implementing a diabetes education program is a major challenge for a multidisciplinary health care team, both in terms of learning from patients and understanding that acquiring knowledge does not necessarily mean changing behavior. There is no single diet plan nor identical menus for all patients. Instead, there are some principles, recommendations, and good examples that provide patients with better guidance in education and lifestyle change [27]. 

Primary limitation of this study is that we did not include the control group. One of the limitations of this study was that its findings could not be generalized to the entire Croatian diabetic population, because it was conducted in one rural geographical area. We recommend that a randomized controlled trial should be carried out to test this intervention in Croatia for patients attending outpatient care. Our findings must also be considered in the context of several limitations, including certain losses, 31 (28%) participants, high mortality, as well as the small sample of participants. Of original participants, 11 (10%) died, (age 65–72 years; myocardial infarction—2 participants, stroke—2 participants, car accident—1 participants, carcinoma—3 participants, and 3 unknown), 7 (6.4%) changed their place of residence (the older population moved in with their children in town), 7 (6.4%) declined to participate in the follow-up, and 6 (5.6%) could not be contacted mostly because of the rural area. Some subjects, 4 (3.6%) who previously used oral hypoglycemics, were transferred to insulin therapy; they had increased glycemia and their medication had changed since their initial involvement in the study and were transferred into another group on insulin therapy and their results of increased glycemia could have influenced the final group results.

## 5. Conclusions

In conclusion, significant improvements were noted for HbA1c, fasting serum glucose, for all patients, and education had a positive effect on glycemic control and adherence to the recommended diet. The results of this study show that, as time passed, two years after the education most participants forgot what they had learned and returned to their usual habits. However, at two-year follow-up, participants showed an increase in glycemia. We think it is necessary to conduct education continuously every six months to once a year, which is consistent with results of the study carried out in Brazil [22], and stress the purpose of the education is to extend life expectancy and reduce complications. 

## Figures and Tables

**Table 1:** Baseline characteristics of studied patients from the questionnaire.

Variable	Insulin Therapy/Insulin + Oral Therapy *n* (%)	Oral Hypoglycemic Therapy *n* (%)	Total *n* (%)	*p* Value
Patients number	56	53	109 (100)	
Gender (*n* (%))				
Male	26 (46)	26 (48)	52 (47.7)	>0.99 *
Female	30 (54)	28 (52)	58 (53.2)	
Age (year/median(range))	57 (48–67)	62 (55–70)	59 (51–68)	0.046 †
Diabetes duration (years/median(range))	11 (5–19)	6 (3–11)	8 (4–16)	0.006 †
Level of education (*n* (%))				
Lower Elementary school grades	6 (10.7)	6 (11.3)	12 (11)	0.868 ‡
Elementary school	17 (30.4)	15 (28.3)	32 (29.3)	
Secondary school	28 (50)	26 (49.1)	54 (49.5)	
Professional education	4 (7.1)	3 (5.7)	7 (6.4)	
College education	1 (1.8)	3 (5.7)	4 (3.6)	
Diabetes type (*n* (%))				
Type 1 diabetes	11 (19.6)	0	11 (10.1)	0.014 *
Type 2 diabetes	40 (71.4)	48 (90.6)	88 (80.7)	
Gestational diabetes	4 (7.1)	2 (3.8)	6 (5.5)	
Steroid diabetes	0	1 (1.9)	1 (0.9)	
Other	1 (1.8)	1 (1.9)	2 (1.8)	
Not known	0	1 (1.9)	1 (0.9)	

Legend: * Fisher’s exact test; † Mann–Whitney test; ‡ χ^2^ test.

**Table 2:** Biometric indices before and after education, and at two-years follow-up.

Values	Median (Interquartile Range)			*p* *
	Before Education	After Education	At Two-Year Follow-Up	
Insulin therapy/Insulin + oral therapy				
Blood glucose (fasted)	8.5 (6.7–12.1)	7.6 (6.3–9.8)	8.1 (6.1–10.63)	0.04
HbA1C (%)	8.2 (7.5–10)	-	7.2 (6.7–8.9)	0.02 †
Systolic pressure	120 (110–140)	120 (110–139)	130 (120–135)	0.53
Diastolic pressure	70 (70–80)	70 (70–80)	80 (70–81.2)	0.003
BMI (kg/m^2^)	30.7 (25.7–33.7)	30.3 (25.6–33.5)	31.62 (23.6–35.7)	0.38
Oral hypoglycemic therapy				
Blood glucose (fasted)	9.0 (7.2–11.7)	8.1 (6.7–9.8)	7.65 (5.95–10)	0.02
HbA1C (%)	8.2 (6.9–8.7)	-	6.9 (6.0–7.5)	0.002 †
Systolic pressure	130 (110–140)	130 (120–140)	130 (120–130)	0.93
Diastolic pressure	80 (60–85)	75 (70–80)	80 (70–80)	0.73
BMI (kg/m^2^)	29.4 (27.4–33.5)	29.3 (27.4–33.3)	29 (26.8–33.7)	0.78

Legend: * Friedman’s test; † Wilcoxon test; HbA1c: glycated hemoglobin; BMI: body mass index.

**Table 3:** Adherence to the recommended diet after education at four weeks and two years compared with pre-education rates.

Adhering to Recommended Diet		Number (%) of Subjects before Education			Total	*p* *
		Never	Periodically	Always		
Insulin therapy/Insulin + oral therapy						
After education	Never	6 (37.5)	0	1 (5.9)	7 (12.5)	0.020
	Periodically	8 (50)	16 (69.6)	4 (23.5)	28 (50)	
	Always	2 (12.5)	7 (30.4)	12 (70.6)	21 (37.5)	
	Total	16 (100)	23 (100)	17 (100)	56 (100)	
At two-year follow-up	Never	1 (11.1)	1 (6.7)	0 (0)	2 (5.4)	0.018
	Periodically	6 (66.7)	13 (86.7)	7 (53.8)	26 (70.3)	
	Always	2 (22.2)	1 (6.7)	6 (46.2)	9 (24.3)	
	Total	9 (100)	15 (100)	13 (100)	37 (100)	
Oral hypoglycemic therapy						
After education	Never	1 (6.3)	1 (3.4)	0	2 (3.8)	0.003
	Periodically	11 (68.8)	26 (89.7)	5 (62.5)	42 (79.2)	
	Always	4 (25)	2 (6.9)	3 (37.5)	9 (17)	
	Total	16 (100)	29 (100)	8 (100)	53 (100)	
At two-year follow-up	Never	2 (14.3)	2 (9.5)	0 (0)	4 (10)	0.048
	Periodically	7 (50)	15 (71.4)	3 (60)	25 (62.5)	
	Always	5 (35.7)	4 (19)	2 (40)	11 (27.5)	
	Total	14 (100)	21 (100)	5 (100)	40 (100)	

Legend: * McNemar–Bowker test.

**Table 4:** Knowledge of foods that increase blood glucose.

Foods that Increase Blood Glucose		Number (%) of Subjects before Education						*p* *
		Beetroot	Bread	Rice	Not Known	Bread and Rice	Total	
Insulin therapy/Insulin + oral therapy								
After education	Bread	0	3 (10.3)	1 (33.3)	1 (8.3)	0	5 (8.9)	<0.001
	Rice	0	0	0	1 (8.3)	0	1 (1.8)	
	Not known	0	0	0	1 (8.3)	0	1 (1.8)	
	Bread and rice	2 (100)	26 (89.7)	2 (66.7)	9 (75)	10 (100)	49 (87.5)	
	Total	2 (100)	29 (100)	3 (100)	12 (100)	10 (100)	56 (100)	
At two-year follow-up	Bread	0	9 (47.4)	1 (50)	2 (33.3)	0	12 (32.4)	0.003
	Rice	0	0	0	1 (16.7)	0	1 (2.7)	
	Bread and rice	2 (100)	10 (52.6)	1 (50)	3 (50)	8 (100)	24 (64.9)	
	Total	2 (100)	19 (100)	2 (100)	6 (100)	8 (100)	37 (100)	
Oral hypoglycemics therapy								
After education	Bread	0	8 (30.8)	0	4 (21.1)	0	12 (22.6)	<0.001
	Rice	0	0	0	1 (5.3)	0	1 (1.9)	
	Cucumber	0	0	0	1 (5.3)	0	1 (1.9)	
	Not know	0	1 (3.8)	0	3 (15.8)	0	4 (7.5)	
	Bread and rice	0	17 (65.4)	0	10 (52.6)	8 (100)	35 (66)	
	Total	0	26 (100)	0	19 (100)	8 (100)	53 (100)	
At two-year follow-up	Beetroot	0	0	0	0	1 (16.7)	1 (2.5)	0.133
	Bread	0	10 (45.5)	0	5 (45.5)	2 (33.3)	17 (42.5)	
	Not know	0	1 (4.5)	0	2 (18.2)	0	3 (7.5)	
	Bread and rice	0	11 (50)	1 (100)	4 (36.4)	3 (50)	19 (47.5)	
	Total	0	22 (100)	1 (100)	11 (100)	6 (100)	40 (100)	

Legend: * Marginal homogeneity test.
